# Advanced analytical methodologies for measuring healthy ageing and its determinants, using factor analysis and machine learning techniques: the ATHLOS project

**DOI:** 10.1038/srep43955

**Published:** 2017-03-10

**Authors:** Francisco Félix Caballero, George Soulis, Worrawat Engchuan, Albert Sánchez-Niubó, Holger Arndt, José Luis Ayuso-Mateos, Josep Maria Haro, Somnath Chatterji, Demosthenes B. Panagiotakos

**Affiliations:** 1 Department of Psychiatry, Universidad Autónoma de Madrid, Madrid, Spain; 2 CIBER of Mental Health, Spain; 3 Hospital Universitario de La Princesa, Instituto de Investigación Sanitaria Princesa (IP), Madrid, Spain; 4 Department of Nutrition and Dietetics, School of Health Science and Education, Harokopio University, Athens, Greece; 5 The Centre for Applied Genomics, Genetics and Genome Biology, The Hospital for Sick Children, Toronto, Ontario, Canada; 6 Parc Sanitari Sant Joan de Déu, Barcelona, Spain; 7 CIBER of Epidemiology and Public Health, Spain; 8 SPRING TECHNO GMBH & Co. KG, Bremen, Germany; 9 Information, Evidence and Research, World Health Organization, Geneva, Switzerland

## Abstract

A most challenging task for scientists that are involved in the study of ageing is the development of a measure to quantify health status across populations and over time. In the present study, a Bayesian multilevel Item Response Theory approach is used to create a health score that can be compared across different waves in a longitudinal study, using anchor items and items that vary across waves. The same approach can be applied to compare health scores across different longitudinal studies, using items that vary across studies. Data from the English Longitudinal Study of Ageing (ELSA) are employed. Mixed-effects multilevel regression and Machine Learning methods were used to identify relationships between socio-demographics and the health score created. The metric of health was created for 17,886 subjects (54.6% of women) participating in at least one of the first six ELSA waves and correlated well with already known conditions that affect health. Future efforts will implement this approach in a harmonised data set comprising several longitudinal studies of ageing. This will enable valid comparisons between clinical and community dwelling populations and help to generate norms that could be useful in day-to-day clinical practice.

Life expectation at birth has been continuously increasing during the last 50 years^1^. The extension of lifespan is not paralleled with extension of healthy lifespan^1^ (healthspan). The improvement in the management of chronic diseases has not been able to impede the accumulation of health deficits with ageing. The cost is extremely high in society and economy, since people are living longer but with multimorbidity and disabilities^2^. With populations worldwide ageing rapidly, due to increasing life expectancies and declines in fertility, one of the major societal challenges in the future will be this ageing population.

Ageing is the process that turns young adults to older adults with waning physical capacities and fitness that increases the risk for illness and death^3^. Ageing is a well-known risk factor for most diseases^4^. It is a multifactorial phenomenon that everyone experiences differently since it involves interactions between biological and molecular mechanisms with the environment^5^. Accordingly, the ageing population is heterogeneous regarding health status which makes extremely important to gain the ability to determine the age-appropriate optimal health and make the distinction between normal ageing changes and health deterioration^5^. Aged individuals commonly have multiple chronic conditions. The simple count of diagnoses or conditions has been found to be associated with mortality or other outcomes^6^,^7^, including high health care expenditures^8^. However, just counting diseases could fail to capture the true impact of health conditions on health status. There is a need to come up with a metric that can quantify the health status of the individuals. This metric could be aggregated to the population level, allowing for comparisons across populations and over time, and being sensitive to change. The International Classification of Functioning (ICF)^9^ and the World Health Organization’s conceptualisation of health status for measurement^10^, should be the conceptual foundations for the creation of this measure of health.

The aims of the present study, and under the context of the Ageing Trajectories of Health: Longitudinal Opportunities and Synergies (ATHLOS) project (EU HORIZON2020–PHC-635316, http://athlosproject.eu/), were to create a metric of health using an Item Response Theory (IRT) approach, which can be used for comparison of the population across the first six waves of the English Longitudinal Study of Ageing^11^ (ELSA) conducted between 2002 and 2012, and to explore determinants of this health score based on socio-demographics. ELSA is one of the longitudinal studies included in ATHLOS, a project which aims to produce a harmonised dataset including European and international longitudinal studies of ageing, in order to identify health trajectories and determinants in ageing populations.

The procedure described in this paper to obtain a global score on health which can be compared across the ELSA waves will be used to create a common metric of health across the longitudinal studies considered in ATHLOS, once the ATHLOS harmonised data set is available. Using a Bayesian multilevel IRT approach, items that vary across studies will be considered together with anchor items and items that vary across waves in order to create a metric of health which allows for comparison across studies and waves of those longitudinal studies. IRT methods can be helpful in developing better health outcome measures and in assessing change over time^12^. Scaling models combining modern IRT, multilevel modelling techniques, and Bayesian estimation techniques have been proposed for deriving information from international survey data^13^. Advanced analytical methodologies in pattern recognition and computational learning, as Machine Learning approaches, can also be employed to explore factors associated with the metric of health.

## Results

### Assessment of unidimensionality at ELSA baseline

From a initial sample of 12,099 participants at ELSA baseline, a total of 175 subjects (1.45%) were excluded from the analysis since they were unable to be interviewed through poor health, or through physical or cognitive disability. Moreover, 18 subjects (0.15%) were also excluded since their information was missing for at least the 25% of the self-reported health questions and measured tests at ELSA baseline. The final sample was comprised of 11,906 participants at ELSA baseline with a mean age of 64.04 years (SD = 10.84) and a 56.0% of women. Regarding visual and hearing difficulties, a total of 409 participants considered their eyesight poor (a 3.44% of the final sample), while 52 (0.44%) were registered or legally blind; 575 participants (4.83%) considered their hearing poor.

A two-step factor analysis approach was conducted to assess the unidimensionality of the self-reported health questions and measured tests found at ELSA baseline. A total of 8,334 (70% of the final sample) participants were assigned to the developmental sample, where an Exploratory Factor Analysis (EFA) was conducted to identify first-order factors; while the remaining 3,572 participants were assigned to the validation sample (30% of the final sample), where a Confirmatory Factor Analysis (CFA) approach was carried out on the validation sample, proposing a model with a second-order structure. A similar proportion of men and women was considered in both developmental and validation samples.

According to the Minimum Average Partial (MAP) test, five was the adequate number of factors to be extracted in the EFA, since the MAP test achieved the minimum value when five factors were extracted. The five factors extracted explained a 63.1% of the total variance, with the first factor explaining a 44.2%. In Table 1, factor loading estimates are shown. Eight of the 45 items showed factor loadings higher than or equal to 0.25 in at least two factors. The goodness-of-fit indices associated to this model were good: Comparative Fit Index (CFI) = 0.96, Tucker-Lewis Index (TLI) = 0.95 and Root Mean Square Error of Approximation (RMSEA) = 0.047 [90% Confidence Interval (CI) = (0.046, 0.048)]. The likelihood ratio test associated to the five-factor model showed a significant result [χ^2^(775) = 6000.68; *p* < 0.001], although this can be influenced by the large sample size.

**Figure 1:**
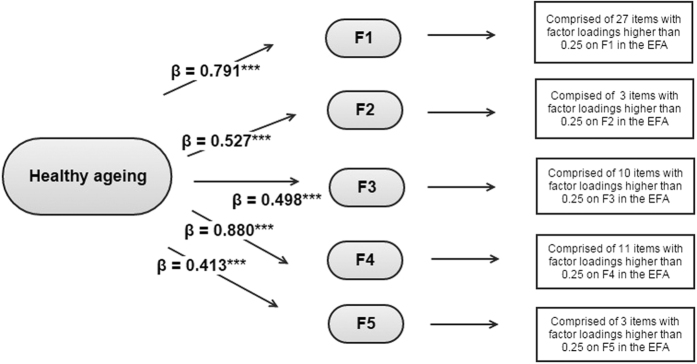
Second-order Confirmatory Factor Analysis conducted over the validation sample at ELSA baseline. ***p < 0.001.

Once identified the first-order factors, a second-order CFA was conducted. The second-order factorial structure comprised the five first-order factors identified in the previous EFA under a general factor which loaded on the first-order factors. Items with loadings higher than or equal to 0.25 in the EFA were assigned to the first-order factors. Note that, in some cases, one item was considered as loading on two different first-order factors. The standardised loadings of the second-order factor on the five first-order factors ranged from 0.41 to 0.88 (Fig. 1). Regarding the first-order factors, the standardised factor loadings were all positive and significant, ranging from: 1) 0.28 to 0.91 in the first factor; 2) 0.85 to 0.89 for the sight items in the second factor; 3) 0.23 to 0.79 in the third factor; 4) 0.26 to 0.87 in the fourth factor; 5) 0.30 to 0.85 in the last factor. The likelihood ratio test associated to the resulting second-order CFA showed a significant result [χ^2^(930) = 4048.56; *p* < 0.001]; on the other hand, the remaining goodness-of-fit indices associated to the model were: CFI = 0.98, TLI = 0.98, and RMSEA = 0.017 [90% CI = (0.016, 0.018)], providing evidence for the assumption of unidimensionality and the creation of a metric of health based on the 45 baseline items considered. As described below, anchor items and items that varied across waves were identified and a Bayesian multilevel IRT approach was used for generating a global health score in each wave.

### Creation of a common metric of health across different ELSA waves

A total of 17,886 subjects (54.6% of women, 36.7% older than 65 at the moment of the first interview) participated in at least one of the six waves of ELSA conducted between 2002 and 2012, and they have been considered in this analysis. A 28.17% of them (*n* = 5038) participated in the first six ELSA waves. Six of the 45 items identified at baseline were only asked in some of the next waves. They were considered items that vary across waves, while the remaining 39 items were considered anchor items.

Four potential models with different characteristics were considered in the Bayesian multilevel IRT approach to generate the metric of health. This global health score was generated for each wave, allowing for the inclusion of anchor items and items that vary across waves (i.e., items that are available at baseline but not in all the remaining waves of the study). Table 2 shows the Expected-A-Posteriori estimation of ability (EAP reliability) and Deviance Information Criterion (DIC) values for each of the four multilevel models initially proposed. The last model showed the highest reliability (EAP reliability = 0.934) and the lowest DIC value (DIC = 984,274.6), and was selected for creating the latent score on health. The specific characteristics of this model were the estimation of item-specific standard deviations of item difficulties and slopes, and the hierarchical prior distribution with estimated item and slope parameters.

The Markov Chain Monte Carlo estimation was run considering 5,000 iterations and 100 burn-in iterations. Latent scores were obtained for each participant in each wave and then transformed into a 0–100 scale, with higher values indicating a better health status. The Intraclass Correlation Coefficient at the wave level was 0.47 [95% CI = (0.28, 0.71)]. Finally, the degree of relationship between the metric generated and a simple sum of the items was assessed, obtaining a high correlation: |*r*| = 0.86.

### Relationship between different factors and health score across the ELSA waves

According to the results of the multilevel regression shown in Table 3, women (*p* < 0.001) and older people (*p* < 0.001 for the two comparisons between older than 80 years and younger than 65, and between people aged 65–79 years and younger than 65) showed lower scores in health, indicating worse health status. Moreover, health status increased as higher the quintile of household wealth. No qualification was related with worse health status (*p* < 0.001).

Participants who had a fall in the previous two years and participants who were not working presented a lower score on health (*p* < 0.001 in both cases). People who were married or in a legal partnership showed higher scores on health than those separated, divorced or widowed (*p* < 0.001); people having a large or moderate social network showed higher scores on health than those having a small social network (*p* = 0.009 and *p* = 0.002, respectively). Regarding the healthy lifestyle behaviours, former and current smokers presented a worse health status than never smokers (*p* < 0.001 in both cases), while those who did not drink (including people who never drank and people who did not drink in the previous 12 months) presented lower scores on health. Physical activity was strongly related to a better health status, with active people presenting the higher scores on health, followed by moderately actives. The significant standard deviation of the intercept across participants (estimate = 8.43, SE = 0.07) justified the use of a random effects part in the multilevel regression model. Besides, the mixed-effect multilevel regression model clearly showed a best fit than a linear model, according to the likelihood-ratio test [χ^2^ (2) = 22,234.96, *p* < 0.0001]. The adjusted McFadden’s pseudo *R*^2^ value associated to the final model was 0.28. In terms of the Akaike Information Criterion (AIC), the final model showed a better performance than the intercept-only model (AIC_full_ = 326,211.3 vs. AIC_intercept_ = 452,778.5), since lower values in information criteria indicate a better fit. A Conditional Intraclass Correlation Coefficient value of 0.72 indicated also a high degree of dependence among observations within the same individual.

When considering age and size of the social network as continuous variables, similar results were found in the mixed-effect multilevel regression model. A higher age was related to a lower health score [Coef. = −0.23; 95% CI = (−0.25, −0.22); *p* < 0.001], while a greater size of the social network was related to a better health status [Coef. = 0.02; 95% CI = (0.01, 0.03); *p* < 0.001]. When considering the model with age and size of the social network as continuous variables, the adjusted McFadden’s pseudo *R*^2^ was also 0.28.

**Figure 2:**
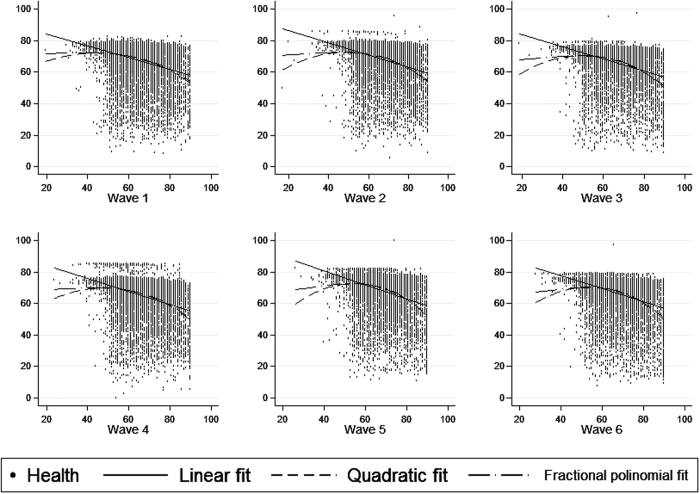
Scatter plots corresponding to the relationship between age (X-axis) and health status (Y-axis) in each ELSA wave. Age was collapsed at 90 years for people older than 90.

In Fig. 2, alternative functional forms (quadratic fit and fractional polynomial fit) to linear fit were explored to describe the relationship between age (measured in a continuous form) and health status. The results obtained were similar across the different fits, suggesting an inverse relationship between age and health status, as expected.

### Machine learning methodologies

A four-class categorisation was considered according to the following groups based on health scores: scores lower or equal to 20, scores higher than 20 and lower or equal than 40, scores higher than 40 and lower or equal to 60, and scores higher than 60. In a preliminary analysis, the Random Forest (RF) classifier outperformed other classifiers in classifying subjects into the four classes proposed, with an average accuracy of 83% (assessed by 10-fold cross-validation), and was employed to model the differences in patterns of factors across the four groups based on health scores

**Figure 3:**
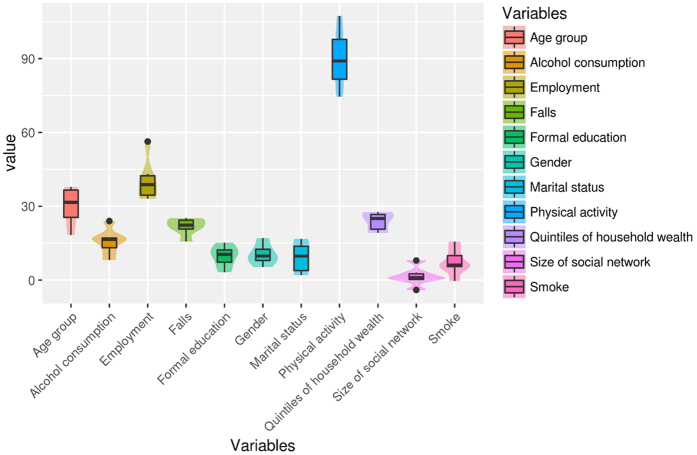
Relationship between different factors and level of health assessed by machine learning method. Mean Decrease Accuracy (MDA) values.

Figure 3 shows the relationship between different factors and level of health. According to the Mean Decrease Accuracy (MDA) estimate, the physical activity factor was the most related factor to the health score (MDA = 89.97). Other four factors highly related to the health score were age group (MDA = 30.18), employment (MDA = 40.60), quintile of household wealth (MDA = 23.91) and falls (MDA = 21.80). Decision Tree (DT) was further applied to identify patterns of the factors driving differences in health score. To follow the well-established strategy of RF, we constructed one hundred DTs, using different sets of variables and samples. Then, an 40% accuracy (the accuracy of a random classifier for four classes is 25%) threshold was applied to select significant patterns from all DTs of each class.

From the extracted patterns, the five most accurate patterns of each class were selected using the DT method and a list of 20 patterns was obtained. Top five factors mostly presented in selected patterns were (between parentheses, the number of occurrences of each factor in the 20 patterns selected): Physical activity (18/20), Quintile of household wealth (13/20), Employment (10/20), Falls (9/20) and Age group (8/20).

### Sensitivity analysis

The metric of health developed by means of the Bayesian multilevel IRT approach showed a higher ability to predict mortality over ten years than the simple count of chronic conditions. After excluding the 360 participants (3.02% of the final sample at baseline) who did not provide consent to linkage to the National Health Service Central Register, a Receiver Operating Characteristics (ROC) analysis adjusting by gender was conducted (considering mortality as gold standard) and the Area Under the ROC Curve (AUC) associated to the metric of health was 0.73 [95% CI = (0.72, 0.75)], while the AUC associated to the number of chronic conditions was 0.56 [95% CI = (0.55, 0.58)]. A higher score on health and a lower number of chronic conditions were related to a lower mortality. A total of 2504 participants who comprised the final sample at baseline (20.03%) died in the following ten years. Finally, the ability to predict institutionalisation over ten years was also assessed, finding similar results. Only 70 of the 5981 participants (1.17%) at ELSA baseline who completed ELSA Wave 6 ten years after were found to be institutionalised. In this case, the AUC associated to the metric of health was 0.72 [95% CI = (0.67, 0.78)], indicating that a higher score on health at baseline was associated with a lower probability of being institutionalised over ten years; while, the AUC associated to the number of chronic conditions was 0.55 [95% CI = (0.47, 0.63)].

Finally, a sensitivity analysis was also conducted in terms of criterion validity over the baseline data. The relationship between the presence of different chronic conditions and health status was assessed, and all the relationships were found significant at a *p*-value lower than 0.001, as shown in Table 4.

## Discussion

In this paper a combination of two data analytical methodologies was applied to create a measure of health in a cohort of older adults. This approach could be considered as a framework for advanced analytical approaches, as well as a paradigm for understanding the ageing process. Here we show that the development of a single metric of health that includes a set of different functioning, mobility, sensorineural, cognitive, emotional aspects is feasible and psychometrically sound. This operationalisation can only be conducted if we conceptualise health as a unidimensional latent construct measured by different domains of human functioning^14^.

A total of 45 items, including self-reported health questions and measured tests, have been identified at ELSA baseline as key questions to obtain the global score on health. Taking as basic items these questions, a mapping was conducted over the remaining ELSA waves, identifying which of these items were available across the remaining waves and which not. The Bayesian multilevel IRT approach employed allows for the inclusion of anchor items (items that are available in all waves) and items that vary across waves. This approach can be extended and applied to the general aims of the ATHLOS project, considering the presence of items that vary across studies together with anchor items and items that vary across waves. The metric of health created could be then employed to compare different trajectories of health across the participants in the longitudinal studies included in ATHLOS.

This metric of health assesses health status conceptualised as a vector of functioning in different domains ranging from simple to complex (e.g., vision, walking, kneeling, Activities of Daily Living, Instrumental Activities of Daily Living). Health has been operationalised according to the notion of ‘health state’ suggested by the World Health Organization: (i) an intrinsic attribute of an individual that can be aggregated to the population level; and (ii) comprising domains of human functioning that describe the actual impact of health conditions on people’s lives^10^. These domains are based on extensive, sophisticated and multi-method studies^15^. In a similar way, Cieza *et al*.^14^ constructed a metric of health operationalised as domains of functioning and based on data directly collected from individuals to compare the health of two populations (English and Americans). The method which they employed was also based on IRT, but the comparison of health scores was conducted in a cross-sectional way between both populations. On the other hand, the metric developed in the present study has taken into account the presence of different sets of health items across ELSA waves, and the procedure described can be applied to surveys with different sets of self-reported health questions. Other studies have shown that it is possible to construct a metric of health based on data directly collected from individuals^16^,^17^,^18^,^19^; however, they focused on the same population or on the same survey, using in both cases a cross-sectional design. The procedure described in the present study can support the comparability of health across different waves and populations.

In the analyses conducted over the ELSA data set, the results obtained in the factor analysis suggested the presence of a general health factor, with five underlying first-order factors. A total of 24 items showed their main loading on the first factor, which could be considered a general factor comprising questions of Functioning and Mobility. The three items related to sight loaded on a same factor, the second one. The third factor was mainly composed of the seven measured tests (cognitive functioning tests and walking speed test), including also secondary loadings for some limitations in Instrumental Activities of Daily Living as making telephone calls, managing money and using maps. The items which showed a higher loading on the fourth factor were mainly those related with energy and sleep, while items about hearing were the questions with highest loadings on the fifth one. The goodness-of-fit-indices obtained for the second-order factor model provided evidence for unidimensionality at ELSA baseline. Then a global health score was created across the different ELSA waves, using the Bayesian multilevel IRT approach.

The methodology employed in the present study to assess unidimensionality of the self-reported health questions and measured tests identified was based on a two-stage factor analysis approach. This two-stage factor analysis approach (which included EFA and CFA) was based on a random split of the overall sample into two groups, a procedure commonly employed in the literature^20^,^21^,^22^,^23^. The essential purpose of factor analysis is the reduction of a set of observable variables to fewer latent variables that share a common variance and are unobservable^24^. However, it is important to note that factor analysis is only one of the several options in data reduction techniques. Although the factor models employed in this study showed a good fit, alternatives based on cross-validation and bootstrap techniques could have been considered. Cross-validation procedures for factor scores have been previously suggested^25^,^26^, while bootstrap methods can be applied to any statistic of interest, even in the multivariate case^27^.

Factor analytic methods have been explored to quantify biological ageing. Using Principal Components Analysis^28^ to characterise the correlation structure of molecules in a biological network, Cohen *et al*.^29^ detected a novel physiological phenomenon (“integrated albunemia”, reflecting the strong implication of measures related to anemia and albumin) which was implicated in ageing. Although little consensus exists regarding optimal methods for calculating biological age, Levine^30^ compared several methods with chronological age on the basis of predictive sensitivity and strength of association with mortality. The methods included in the comparison were principal component analysis, multiple linear regression, and a mathematical algorithm developed by Klemera and Doubal^31^, which was based upon minimising the distance between *m* regression lines and *m* biomarker points, within an *m*-dimensional space of all biomarkers. The Klemera and Doubal’s method showed the best performance in the Levine’s study^30^ in terms of predicting mortality.

At this point, it is important to note that the present study has defined a metric of healthy ageing based on self-reported health questions and measured tests, instead of biomarkers. However, alternative approaches conducted over biomarker data could be used in future studies to assess their performance and overcome potential limitations of methods based on factor analysis. These alternatives include: (a) algorithms developed to predict chronological age in a reference sample, which are then used to calculate biological age^30^,^32^; and (b) data reduction techniques based on statistical distance of individuals from some normative reference population. Li *et al*.^33^, employing a procedure developed by Cohen *et al*.^34^ used a younger population as the reference group (healthier reference population) and calculated the Mahalanobis distance^35^ from each participant to the centroid of the reference group in the multivariate biomarker space.

The health score developed in this study showed a good correlation with well-established health determinants, according to the results obtained in the mixed-effects multilevel regression model. Females, poor people and those who are less educated, had worse health status than those with higher income and education level. This was also observed by Cieza *et al*. when they used the same conceptual approach^14^. We have also found that people of older age, smokers, people that are either divorced or widowed, have falls, are physically inactive and those with less social interactions have worse health status. Surprisingly, we have found that people consuming alcohol have better overall health. A recent study^36^ using data from ELSA waves revealed that older adults with poor Self Rated Health (SRH) were not drinking as much as adults with good SRH, suggesting that frequency of drinking was not a determinant of health status but rather a signifier of good health. Age is a well-documented risk factor for most human diseases. This is a well-established knowledge sourcing from several epidemiological studies but it has also been documented on genome level. A recent network gene analysis showed that genes regulating ageing process are more likely to be related to some kinds of diseases^37^.

Inequity in physical and social environment that people are living in is responsible for the heterogeneity observed in older age. This can be either a direct effect of the environment or through barriers or incentives that are defining behavioural patterns, opportunities in life and decision making^38^. People with the lowest income show a lower peak of functioning, a trend that remains during the lifespan. Three out of four people by the age of 68 experience a disability with most of them living in the poorer neighborhoods^38^.

Physical activity engagement provides multiple benefits in older people. Moderate intensity activities, for 150 min/week, decrease mortality by 31% in older people while they improve their physical and mental capacities, reduce their risk for chronic diseases and expand their social networks and links^39^. As it has been recently shown functional status, cognitive function and self-reported estimation of physical health correspond to biological measures of ageing^40^. The same approach has been also used as an instrument by Cieza *et al*.^14^ in order to compare health status between an American and an English cohort. They found that the outcome when comparing cohort overall health using this technique differed from that when the comparison was made with health conceived only by counting chronic health conditions^14^. This result provides new perspectives for using such an approach. This kind of health instrumentalisation may reveal differences in health status between populations or even across populations that have been hidden or were believed to exist. More accurate health status evaluation and description of its evolution may arise giving public health policy makers useful information about the real challenges people are dealing with while they age.

Healthy ageing is a continuous phenomenon that can be described by several hypothetical trajectories for functional abilities and intrinsic capacities. These trajectories reflect the interaction between individuals and the environment they are living in^38^. By creating a health score that encompasses functioning items along with measured tests and information about diseases we can follow this process through time and describe realistic health trajectories that correspond to population as well as to personal level. In fact, this health score is constructed in a way that can be used for comparison between different epidemiological cohorts, coming from different countries and in order to describe ageing trajectories among populations. This approach will be extended to other cohorts as part of the ATHLOS project. The usefulness this metric of health is clear. Firstly, it will serve as a concise and sound tool that can be used to comprehensively compare health between different countries, different regions and different times. Secondly we can track in time evolution of overall health of each individual participating in different waves of epidemiological studies. This will provide us the chance to describe ageing trajectory patterns that are real and not just theoretical models. Additionally, the measure can be used to examine different individuals following different trajectories over the course of their lifetime and address the different determinants of these varying trajectories.

The methodology employed in the present study, the approach and the metric of health developed can be extended to the longitudinal studies included in the ATHLOS project. The main aims in ATHLOS are: to achieve a better understanding of ageing by identifying patterns of healthy ageing pathways or trajectories, the determinants of those patterns, the critical points in time when changes in trajectories are produced, and to propose timely clinical and public health interventions to optimise healthy ageing. The health metric created will allow for the comparison of health scores across populations, studies and waves, since the Bayesian multilevel IRT approach presented in this study takes into account the presence of anchor items and items that vary across waves and studies.

To further strengthen the information extracted using traditional statistical methods on the ELSA data, Machine Learning methods were used to identify patterns in the data which characterise individuals at various levels of health. After providing the designated algorithms with inputs of selected factors (including socio-demographics, socio-economics and healthy lifestyle behaviours), each sample was categorised into one of four classes, each one representing a range of health scores. Regarding the relationship between different factors and the health score, the results obtained using Machine Learning methods were consistent with those previously found using a mixed-effect multilevel regression approach. It should be taken into account that the continuous health score was categorised in four groups based on accuracy and performance of the Random Forest algorithm and this could be considered as a potential limitation. Although the non-parametric Machine Learning methods had a secondary role in the analyses conducted over the ELSA data set (they were mainly used to check the results previously found using a mixed-effects regression model), they will be one of the main statistical techniques to be applied in the ATHLOS project. They will be employed to complement traditional statistical techniques and generate classes of healthy ageing pathways with different algorithms.

There are some limitations of this study. For example, only one source, the ELSA study, has been used. Its application to a range of data sets from different epidemiological studies will need to be shown when ATHLOS harmonised data set is available. In addition, possible missing values in the items considered to construct the metric of health could lead to underrepresent people with worse health status, and the results related to the determinants of health status could be biased. Moreover, the ELSA sample was designed to be focused on people aged 50 and over. In a similar case to other longitudinal studies considered in the ATHLOS project, it includes a group of subjects aged between 18 and 49 years for comparison purposes. The use of the procedure employed in the present study to create a metric of health in the ATHLOS harmonised data set will not allow only for comparisons across waves and populations of different longitudinal studies, but also for comparisons among young adults, middle-aged adults and older adults. To study the process of ageing in younger cohorts and to quantify differences among healthy individuals in their pace of ageing can help to develop effective strategies to prevent age-related diseases before they onset^41^.

As a sensitivity analysis, the ability of the metric of health to predict mortality and institutionalisation was assessed. A higher score on health at baseline was related to a lower mortality and a lower probability of being institutionalised in the following ten years. Although the associated AUC value was far from indicating a perfect predictive ability, the health score created showed a better performance than the simple count of chronic conditions. Moreover, the presence of each of these chronic conditions was found to be significantly associated with the health score (after conducting a multiple linear regression model), providing some evidence for the criterion validity of the metric of health created. Further studies are needed correlating healthy ageing measurements with other measures of morbidity and mortality (considering different time periods), use of health care services, and geriatric syndromes such as frailty. Clinical information relevant for health care providers could be incorporated into such a measure and can then be used in everyday practice to benchmark clinical subjects to community dwelling populations. This measure can also be used to quantify the effects of clinical and public health interventions and thereby guide individual patient management and public health policy and monitor these over time.

## Methods

### Sample and Study design

The ELSA study began in 2002 and is a biannual, longitudinal and nationally representative survey that focuses on adults aged 50 and over. The data and associated documentation corresponding to the first six ELSA waves considered in this study are all available at the UK Data Service data catalogue [https://discover.ukdataservice.ac.uk/]. ELSA data collection was conducted by means of a face-to-face interview (a computer–assisted personal interview followed by a self-completion questionnaire). All the specific details about sample design, data collection and characteristics of the sample interviewed in each wave are available at the ELSA project website [ https://www.elsa-project.ac.uk/]. Participants at ELSA baseline were recruited from the Health Survey for England (HSE), an annual cross-sectional survey that was designed to monitor the health of the general population, in 1998, 1999 and 2001. Eligibility criteria were: (a) membership of a participant household from HSE in which at least one person had agreed to follow-up; (b) born before 1 March 1952; and (c) living in a private household in England at the time of the first wave of fieldwork. Moreover, in addition to the target sample, partners who were younger than 50 years and those who had joined the household since HSE were invited for interview. Sample members who were unable to be interviewed through poor health, or through physical or cognitive disability, were not considered for the analyses conducted in the present manuscript since information about self-reported health questions and measured tests was not available. All methods were carried out in accordance with relevant guidelines and regulations. All participants in ELSA have given informed consent. Ethical approval for all the ELSA waves was granted from the National Research Ethics Service (MREC/01/2/91). All the details about the ELSA cohort profile are described in detail elsewhere^11^.

### Measures

Self-reported health questions and measured tests were identified to operationalise health status in terms of domains of human functioning, as Walking, Sight, Hearing, Balance, Dizziness, Memory, Orientation in time, Cognition, Pain, Energy, Sleep, Incontinence, Mobility, and limitations in Activities of Daily Living (ADLs) and Instrumental Activities of Daily Living (IADLs). Variables that fit with the proposed minimal generic set of ICF^42^ as well as some measured tests and information about specific diseases that can affect functioning, were employed for the purpose of creating a global health score. This is consistent with World Health Organization’s conceptualization of health for purposes of measurement^10^. A total of 45 items related to self-reported health questions or measured tests, identified at ELSA baseline, were considered as starting point for creating a global health score which can be compared across waves. The variables include self-reported health questions (with questions as ‘How would you rate your memory at the present time?’, ‘Much of the time during the past week, could you not get going?’, or ‘Are you often troubled with pain?’), limitations in ADLs (e.g., eating, bathing, dressing) and IADLs (e.g., managing money, housekeeping, preparing food), and measured tests for walking speed, memory, orientation in time and cognition. Since the majority of the items were negative, the response options for the remaining questions were recoded so that higher values indicated worse health. The overall self-reported health question was excluded from the measure.

Regarding the 45 items identified at baseline, 38 of them were self-reported questions (consisting of impairments in body functions, ADLs and IADLs) and the remaining seven health variables were selected from the measured tests. Walking speed was calculated by dividing distance by the mean time of the two trials. A lower time in the walking speed test indicated a higher speed. The sample was divided into three groups: high (<25^th^ percentile of the distribution), medium (times in the interquartile range of the distribution) and low (>75^th^ percentile of the distribution). Cognitive functions were assessed by immediate and delayed recall of 10 common nouns, verbal fluency, processing speed, a numeracy test consisting of six numeracy questions, and an orientation test consisting of reporting day, date, month and year. For orientation in time, the original variable with five possible values was recoded in “No difficulty” (people who responded correctly the four questions) and “Some difficulties” (people who failed some of the four questions). For the remaining five variables obtained from the measured tests, the procedure described by Cieza *et al*.^14^ was employed and the sample was divided into three groups: high (>one standard deviation (SD) above the mean), medium ( ± one SD around the mean) and low (<one SD below the mean).

#### Social determinants

Gender, age group (<65 years, 65–79 years, older than 80 years), educational attainment (no qualification, some formal education), marital status (married or in a legal partnership, never married, separated/divorced/widowed) and employment status (being in work, not in work) were assessed. The quintile of household wealth was also obtained; household wealth has been identified as the most accurate indicator of long-term socio-economic circumstances in ELSA^43^ and was assessed including savings and investments, value of any property or business assets, net of debt, excluding pension assets.

#### Healthy lifestyle behaviours

Self-reported health behaviours included smoking status (*current, former* and *never smoker*) and alcohol consumption. Based on information about the frequency of drinking in the previous 12 months, three categories were created for alcohol consumption: *does not drink, twice a week or less* and *more than twice a week*. Non-drinkers were combined with participants who did not drink at all in the previous 12 months to form the category *does not drink*. Moreover, physical activity was assessed by asking participants how often they engaged in vigorous, moderate or mild physical activity. Three groups were the created: *active* (more than once a week), *moderately active* (once a week or 1–3 times/month) and *inactive* (hardly ever/never).

#### Other measurements

Participants were asked if they had at least one fall in the previous two years. The size of the social network was measured asking the participants for the number of children/friends/other immediate family (e.g., siblings or cousins) they thought they had a close relationship with. A three-category variable was created, with *small social network* indicating fewer than 5 people in the social network, *moderate social network* indicating 5–9 people, and *large social network* representing 10 or more people in the social network.

#### Chronic conditions and mortality

A list of eight chronic conditions were assessed and used in the present study for sensitivity analysis. Participants were asked whether a doctor had ever told them they had any of the eight following conditions: (1) chronic lung disease (such as chronic bronchitis or emphysema); (2) asthma; (3) arthritis (including osteoarthritis, or rheumatism); (4) osteoporosis; (5) stroke; (6) cancer or a malignant tumor; (7) diabetes; and (8) any emotional, nervous or psychiatric problems. It was also assessed if participants at ELSA baseline were in an institution ten years after (at the moment of Wave 6).

Mortality checking was carried out for ELSA sample members who provided consent to linkage to the National Health Service Central Register at ELSA Wave 1. The mortality status used was updated at February 2012 (ten years after the baseline evaluation).

### Statistical analysis

The unidimensionality of the self-reported health questions and measured tests identified at ELSA baseline was assessed by means of a two-stage methodology based on factor analysis. An Exploratory Factor Analysis (EFA) was conducted on the developmental sample (70% of the sample) to detect the latent structure among the items related to self-reported health questions or measured tests. An approach for dealing with missing data was employed, using the Full Information Maximum Likelihood^44^ and the Expectation-Maximization (EM) algorithm^45^. The EM algorithm can be used for computing maximum likelihood estimates from incomplete data. In the present study, it was applied to the factor analysis for dealing with potential missing values across the 45 self-reported health questions and measured tests considered (allowing a 25% of missing values across these 45 items, as maximum). Minimum Average Partial (MAP) test^46^ was employed to select the number of factors to extract and Geomin oblique rotation solution for correlated factors was used. A second-order Confirmatory Factor Analysis (CFA) was then applied over the validation sample (30% of the sample) in order to obtain evidence for using a global score on health. The factors identified in the EFA were considered as first-order factors, under a second-order factor structure, assigning to each factor the items with higher loadings in the EFA. The Robust Weighted Least Square estimator for categorical variables was used in both EFA and CFA. Goodness-of-fit of the model was evaluated according to the standard recommendations^47^,^48^. Values of the Comparative Fit Index (CFI) and Tucker-Lewis Index (TLI) above 0.90 were considered to represent an adequate fit; values of Root Mean Square Error of Approximation (RMSEA) less than 0.05 indicated a good fit^49^. Additionally, the likelihood ratio test was reported; however, since the chi-square statistic is sensitive to sample size^50^, statistically significant values (when the sample size is large) might erroneously imply a poor data-to-model fit^51^. Once evidence of unidimensionality was obtained, a mapping was conducted across the remaining ELSA waves considered in this study in order to identify common items across the six waves and items that vary across waves. Then, a metric of health was created across waves considering all the items that were available at baseline and assuming missing values to be random.

In the present study, a Bayesian multilevel IRT was conducted to create the metric of health for the different ELSA waves, using the Markov Chain Monte Carlo approach to estimate all parameters simultaneously^52^. Using this approach, individual latent scores on health can be compared across waves, allowing for different sets of items in each wave. Item parameters were allowed to vary across waves. For identification purposes, the mean of all item slopes was set to one. Random effects for the different waves were included and, in each wave, participants with more than a half of the items missing were excluded. Four potential Bayesian multilevel IRT models were considered initially in order to identify which of the multilevel models provided a better fit to the data and the model selected was then used to calibrate the items and create the metric of health. The characteristics of the four models considered were based on the variance components to be estimated: (1) no intercept variance, no slopes; (2) item-wise intercept variance, no slopes; (3) homogeneous intercept variance, no slopes; (4) intercept variance and slope variances–hierarchical item and slope parameters. The final multilevel IRT model was selected according to the reliability of the Expected-A-Posteriori (EAP) estimation of ability and the value of the Deviance Information Criteria (DIC), which is an indicator of the model fit and of the model complexity. Higher values in EAP reliability indicated a higher reliability of the test (i.e., the construct underlying the self-reported health questions and measured tests considered), while lower DIC values indicated a better model fit. The latent trait score was transformed into a 0–100 scale, with higher values indicating better health. Finally, a mixed-effects multilevel regression was conducted in order to assess the effect of selected socio-demographics, socio-economics, healthy lifestyle behaviours and other factors as falls or size of the social network, on the health score. An alternative model was conducted considering age and size of the social network in a continuous form and adjusting for the remaining factors. In a descriptive way, the relationship between age and health status was assessed by means of scatter plots in each wave. The following fits were illustrated: linear, quadratic and based on fractional polynomials.

Machine learning technique was used to assess concurrent validity and identify factors and their patterns that may be related to the metric of health. We applied the well-known classifier^53^ and the explainable classifier^54^ to extract the importance of factors and their patterns related to the health score. A Random Forest (RF) classifier was built to model the differences in patterns of factors across groups based on the health score. Accuracy was estimated based on how well the model correctly put samples into their groups. A preliminary analysis was previously conducted to compare RF with other state-of-the-art classifiers [e.g., Support Vector Machine (SVM), Neural Network (NN), Decision Tree (DT), Repeated Incremental Pruning to Produce Error Reduction (RIPPER) and Super Learner]. Random Forest (RF) is a tree-based ensemble classifier which combines hundreds of decision trees, constructed by means of different sets of samples and factors. Hence, the model is not over-fitted to the training data and well-suited for practical study^53^. A RF model was built in order to assess the importance of each factor using Mean Decrease Accuracy (MDA). MDA represents the change in the level of accuracy when a particular variable is removed, with higher values indicating that the variable is highly related to the outcome considered. In addition to the MDA value in the RF classifier, Decision Tree (DT), a tree-like-structure classifier, was applied to extract patterns associated with health scores, making a prediction based on a set of if-else conditions. To construct a DT, the relevance of given variables was assessed using the entropy-based method (i.e. information gain, gain ratio, etc). At each split of the tree, a variable having highest entropy was picked to generate an if-else condition to expand the knowledge of the tree. The tree expansion stopped once the termination condition was reached.

A sensitivity analysis was conducted to determine the ability of the metric of health developed in the present study and the simple count of chronic conditions to predict mortality over ten years. The number of chronic conditions was obtained by a sum of the items presented in the list of chronic conditions described in the Measures subsection. Receiver Operating Characteristics (ROC) curves and the Area Under the ROC Curve (AUC) were employed. The AUC assessed the ability of the two measures (the metric of health and the number of chronic conditions) to correctly classify both those who survived and those who died. AUC values can range from 0.5 (representing no predictive ability) to 1 (representing perfect predictive ability). A comparison of AUC values was conducted^55^, after adjusting for gender. For this analysis, health status and number of chronic conditions were taken at baseline, while mortality in the 10-year follow-up was considered as gold standard. A similar analysis was also conducted to assess institutionalisation over ten years. Finally, the relationship between the presence of each of the chronic conditions considered and health score was assessed by means of a multiple linear regression model, employing ELSA baseline data and controlling for gender, age and formal education.

Data management and general analyses were carried out with Stata^56^. Mplus^57^ was employed to conduct the factor analyses, while the *sirt* package^58^ in R^59^ was employed for the Bayesian multilevel IRT approach. For the classification tasks related to machine learning, *randomForest*^60^ and *rpart* packages^61^ in R were used to build the models.

## Additional Information

**How to cite this article:** Caballero, F.F *et al*. Advanced analytical methodologies for measuring healthy ageing and its determinants, using factor analysis and machine learning techniques: the ATHLOS project. *Sci. Rep.* **7**, 43955; doi: 10.1038/srep43955 (2017).

**Publisher's note:** Springer Nature remains neutral with regard to jurisdictional claims in published maps and institutional affiliations.

## Figures and Tables

**Table 1:** Five-factor solution corresponding to EFA conducted on the developmental sample at ELSA baseline (n = 7756): factor loading estimates after Geomin rotation.

Variable	F1	F2	F3	F4	F5
*Difficulty climbing one flight stairs without resting*	**0.87**	0.03	0.13	−0.02	−0.07
*Difficulty walking 100 yards*	**0.89**	0.01	0.17	−0.01	−0.08
*Difficulty pulling or pushing large objects*	**0.85**	0.02	−0.01	0.06	−0.01
*Difficulty lifting or carrying weights over 10 pounds*	**0.83**	0.01	0.01	0.06	−0.02
*By yourself and without using any special equipment, how much difficulty do you have walking for a quarter of a mile?*	**0.82**	0.01	0.22	0.03	−0.05
*Difficulty doing work around house and garden*	**0.83**	0.04	0.04	0.07	−0.05
*Difficulty walking across a room*	**0.89**	0.01	0.12	−0.06	−0.08
*Difficulty getting in and out of bed*	**0.84**	−0.02	−0.12	0.08	0.03
*Difficulty shopping for groceries*	**0.75**	0.04	0.16	0.14	−0.11
*Difficulty bathing or showering*	**0.80**	−0.01	0.10	0.04	0.01
*Difficulty climbing several flights stairs without resting*	**0.85**	−0.01	0.05	−0.04	0.02
*Difficulty stopping, kneeling or crouching*	**0.90**	−0.04	−0.04	−0.12	0.11
*Difficulty using the toilet, including getting up or down*	**0.86**	0.02	−0.05	−0.07	0.01
*Difficulty dressing, including putting on shoes and socks*	**0.86**	−0.04	−0.06	−0.04	0.01
*Difficulty getting up from chair after sitting long periods*	**0.86**	−0.04	−0.11	−0.08	0.16
*Difficulty preparing a hot meal*	**0.65**	0.04	0.17	0.18	−0.06
*Difficulty reaching or extending arms above shoulder level*	**0.65**	0.02	−0.09	0.09	0.07
*Difficulty sitting 2 hours*	**0.73**	−0.02	−0.20	0.01	0.13
*How often do you have problems with keeping your balance when you are walking on a level surface?*	**0.51**	0.04	0.06	**0.25**	0.13
*Difficulty eating, such as cutting up food*	**0.65**	0.05	0.08	0.05	−0.02
*Difficulty picking up 5p coin from table*	**0.64**	0.01	−0.03	−0.01	0.14
*How often do you have problems with dizziness when you are walking on a level surface?*	**0.28**	0.05	−0.01	**0.37**	0.16
*Difficulty making telephone calls*	0.04	0.15	**0.25**	**0.33**	**0.30**
*Difficulty managing money, e.g., paying bills, keeping track expenses*	0.07	0.07	**0.31**	**0.46**	0.12
*Difficulty taking medications*	**0.28**	0.06	0.21	**0.31**	0.04
*Do you find it difficult to follow a conversation if there is background noise, such as TV, radio or children playing (using a hearing aid as usual)?*	0.02	−0.02	0.03	0.06	**0.83**
*Is your hearing (using a hearing aid as usual) …?*	−0.01	0.18	0.06	−0.01	**0.69**
*How would you rate your memory at the present time?*	−0.11	0.11	0.11	**0.27**	0.22
*Difficulty using map to figure out how to get around strange place*	0.15	0.14	**0.25**	**0.27**	0.07
*Is your eyesight (using glasses or corrective lens) as usual … ?*	0.01	**0.89**	−0.01	0.01	0.01
*How good is your eyesight for seeing things at a distance, like recognising a friend across the street (using glasses or corrective lens as usual)?*	0.02	**0.85**	0.01	−0.01	0.01
*How good is your eyesight for seeing things up close, like reading ordinary newspaper print (using glasses or corrective lens as usual)?*	0.01	**0.87**	−0.02	0.01	0.01
*Are you often troubled with pain?*	**0.69**	−0.05	−0.16	0.10	0.08
*I feel full of energy these days*	**0.36**	−0.01	0.01	**0.41**	0.04
*Much of the time during the past week, have you felt that everything you did was an effort?*	0.05	−0.07	0.03	**0.86**	−0.05
*Much of the time during the past week, could you not get going?*	−0.03	−0.08	−0.05	**0.94**	−0.03
*Much of the time during the past week, has your sleep been restless?*	0.10	−0.01	−0.15	**0.54**	−0.02
*During the last 12 months, have you lost any amount of urine beyond your control?*	**0.32**	0.06	0.07	0.16	0.04
*Delayed recall (measured test)*	−0.01	−0.16	**0.89**	−0.02	0.06
*Immediate recall (measured test)*	0.01	−0.10	**0.83**	−0.03	0.07
*Verbal fluency (measured test)*	0.01	0.01	**0.59**	0.06	0.01
*Numeracy test (measured test)*	0.02	0.03	**0.47**	0.12	−0.05
*Processing speed (measured test)*	0.08	0.03	**0.42**	0.01	0.07
*Orientation in time (measured test)*	0.01	0.02	**0.34**	0.06	0.06
*Walking speed (measured test)*	**0.45**	0.04	**0.31**	0.09	−0.14

In bold, factor loadings higher than or equal to 0.25.

**Table 2:** Reliability of the Expected-A-Posteriori (EAP) estimates and Deviance Information Criterion (DIC) values associated to four different Bayesian multilevel IRT models.

Model	EAP reliability	DIC value
1) No intercept variance, no slopes	0.903	1,194,740
2) Itemwise intercept variance, no slopes	0.920	1,112,449
3) Homogeneous intercept variance, no slopes	0.906	1,112,414
4) Intercept variance and slope variances–hierarchical item and slope parameters	**0.934**	**984,274.6**

In bold, highest EAP reliability value and lowest DIC value.

**Table 3:** Mixed-effect multilevel regression to assess the relationship between different factors and health score.

Variables	Coefficient (95% CI)	|z|	*p*
Fixed part			
Intercept	57.27 (56.49, 58.05)	143.91	<0.001
Gender (Ref. Male)	−1.54 (−1.87, −1.22)	9.31	<0.001
Age group (Ref. <65 years)			
* 65–79 years*	−0.63 (−0.83, −0.42)	5.96	<0.001
* 80*+ *years*	−4.43 (−4.79, −4.08)	24.36	<0.001
Quintile of household wealth (Ref. 1^st^ quintile)			
* 2*^*nd*^ *quintile*	2.46 (2.13, 2.79)	14.51	<0.001
* 3*^*rd*^ *quintile*	3.62 (3.28, 3.97)	20.37	<0.001
* 4*^*th*^ *quintile*	4.48 (4.11, 4.84)	24.21	<0.001
* 5*^*th*^ *quintile*	5.03 (4.64, 5.41)	25.49	<0.001
Formal education (Ref. No qualification)	0.84 (0.55, 1.13)	5.67	<0.001
Marital status (Ref. Married)			
* Never married*	0.18 (−0.41, 0.78)	0.61	0.54
* Separated/divorced/widowed*	−1.11 (−1.39, −0.83)	7.77	<0.001
Falls (Ref. No)	−1.61 (−1.77, −1.45)	19.30	<0.001
Smoke (Ref. Never smoker)			
* Former smoker*	−1.48 (−1.77, −1.18)	9.81	<0.001
* Current smoker*	−0.99 (−1.37, −0.61)	5.09	<0.001
Alcohol consumption (Ref. Not drinking)			
* Twice a week or less*	2.68 (2.40, 2.96)	18.69	<0.001
* More than twice a week*	3.36 (3.03, 3.68)	20.07	<0.001
Physical activity (Ref. Inactive)			
* Moderately active*	5.38 (5.04, 5.72)	30.80	<0.001
* Active*	8.20 (7.90, 8.51)	52.77	<0.001
Employment (Ref. Not in work)	2.88 (2.67, 3.09)	26.65	<0.001
Size of the social network (Ref. Small)			
* Moderate*	0.25 (0.09, 0.41)	3.07	0.002
* Large*	0.27 (0.07, 0.47)	2.62	0.009
**Random part (Level = ID)**	**Estimate**	**SE**	**95% CI**
Standard deviation of random intercept	8.43	0.07	(8.30, 8.57)
Standard deviation of random effect corresponding to Wave	1.00	0.02	(0.95, 1.04)
Standard deviation of the level-1 residuals	5.28	0.02	(5.23, 5.33)

**Table 4:** Multiple linear regression model to assess the relationship between the presence of different chronic conditions and the health score created.

Variables	Coefficient (95% CI)	*p*	|β|
Intercept	85.32 (83.86, 86.78)	<0.001	—
Chronic lung disease (Ref. No)	−6.95 (−7.75, −6.15)	<0.001	0.13
Asthma (Ref. No)	−3.01 (−3.61, −2.40)	<0.001	0.08
Arthritis (Ref. No)	−8.66 (−9.09, −8.24)	<0.001	0.31
Osteoporosis (Ref. No)	−7.68 (−8.60, −6.75)	<0.001	0.13
Stroke (Ref. No)	−9.38 (−10.48, −8.27)	<0.001	0.13
Cancer or a malignant tumour (Ref. No)	−1.70 (−2.51, −0.89)	<0.001	0.03
Diabetes (Ref. No)	−4.50 (−5.35, −3.66)	<0.001	0.08
Psychiatric problems (Ref. No)	−4.67 (−5.41, −3.94)	<0.001	0.10

Sensitivity analysis over the ELSA baseline. The analysis was adjusted for gender, age and formal education.
